# Exploring the bidirectional relationship between sleep disorders and atrial fibrillation: implications for risk stratification and management

**DOI:** 10.1186/s43044-024-00524-z

**Published:** 2024-07-30

**Authors:** Ikponmwosa Jude Ogieuhi, Oshomoh Mark-Anthony Ugiomoh, Mishael Awe, Maham Khan, Julia Mimi Kwape, Deborah Akpo, Barkavi Thiyagarajan, Nnokam Prayer Nnekachi

**Affiliations:** 1 Siberian State Medical University, Tomsk, Russia; 2 Medical Academy Named After S I Georgievskiy Crimean Federal University Named After V I Vernadsky, Simferopol, Russia; 3 Fatima Jinnah Medical University, Lahore, Pakistan; 4 University of Botswana School of Medicine, Gaborone, Botswana; 5 State Neuropsychiatric Hospital, Nawfia, Anambra State, Nigeria; 6 Far Eastern Federal University, Vladivostok, Russia; 7 Ivano Frankivsk National Medical University, Ivano Frankivsk, Ukraine

**Keywords:** Sleep, Atrial fibrillation, Insomnia, Arrhythmia, Metabolic syndrome, Apnea, BMI

## Abstract

**Background:**

Atrial fibrillation (AF) is characterized by the absence of p-waves on ECG and irregular rhythm. It often presents with palpitations either palpitations may occur acutely over a short period or intermittently over several years. Other cardinal symptoms of atrial fibrillation include fatigue, dyspnea, and lightheadedness; it is important however to note that most affected individuals are asymptomatic. Concurrently, sleep disorders such as obstructive sleep apnea (OSA), insomnia, narcolepsy, and circadian rhythm disorders which are a group of conditions associated with the body’s internal clock that affect the timing of sleep and alertness, are raising concerns due to their potential associations to arrhythmias. This review explores the bidirectional relationship between AF and sleep disorders, highlighting their implications for risk stratification and management strategies.

**Main body:**

The narrative approach of this review synthesizes evidence from numerous studies obtained through meticulous literature searches. Specific sleep disorders with a bidirectional relationship with AF are the focus, with scrutiny on the prevalence of this connection. The examination delves into the pathophysiology of sleep-related autonomic dysregulation and inflammation, emphasizing potential management modalities. Various meta-analysis cohorts have highlighted a strong connection between sleep disorders and atrial fibrillation (AF). Patients with sleep disorders, especially OSA, have a higher likelihood of developing AF, and conversely, those with AF are more prone to sleep disorders. This impact is not limited to development, as sleep disorders also contribute to the progression of AF, with AF, in turn, negatively impacting sleep duration and quality. Sleep disorders may play an important role in atrial remodeling as well as electrophysiological abnormalities, rendering the atrial tissue more susceptible to arrhythmogenesis. The narrative review suggests that treating sleep disorders could not only improve sleep quality but also reduce risk factors associated with atrial fibrillation. The effective management of sleep disorders emerges as a potential challenge in preventing and treating atrial fibrillation.

**Conclusion:**

In conclusion, this narrative study highlights the bidirectional relationship between sleep disorders and atrial fibrillation. There is a positive correlation, affecting the development, progression, and management of atrial fibrillation. The detrimental impact of sleep disorders on atrial remodeling and electrophysiological abnormalities underscores the significance of their diagnosis and treatment. Education about the importance of sleep and the benefits of sleep disorder treatment becomes imperative for patients with AF and sleep disorders.

## Background

Sleep disorders are a group of conditions that disturb normal sleep patterns [1]. Sleep disorders can be caused by mental health disorders, stress, irregular sleep schedules, medications as well as lifestyle habits. The International Classification of Sleep Disorders (ICSD) helps provide a standardized classification for sleep disorders [2]. These are insomnia, sleep-disordered breathing, central disorders of hypersomnolence, circadian rhythm sleep–wake disorders, parasomnias, and sleep-related movement disorders. The prevalence varies based on the specific condition. These sleep problems can be acute or chronic and affect both the pediatric and adult populations.

In the USA, studies have shown that the prevalence of sleep disorders in the general population ranges from 20 to 41.7% [3].

It can be noted that across the world, the most common sleep disorder is insomnia, followed by sleep apnea [4]. Several studies have shown that insomnia generally tends to be higher in females, while sleep-disordered breathing is higher in the elderly.

Atrial fibrillation is the most common type of cardiac arrhythmia [5]. The current global prevalence of atrial fibrillation is approximately 60 million cases and is expected to increase in the coming years [6].

There are numerous risk factors predisposing to the occurrence of atrial fibrillation ranging from hypertension, and endocrine disorders such as hyperthyroidism, and diabetes to neurologic disorders such as stroke. Lifestyle habits that increase the risk of atrial fibrillation include increased alcohol consumption and a sedentary lifestyle. Sleep disorders also have a causal relationship with atrial fibrillation. It is highly associated with obstructive and central sleep apnea [7].

Notably, patients with sleep apnea have four times the risk of developing AF as over time the sleep apnea leads to the onset of risk factors such as hypertension and diabetes known to cause AF [8]. The prevalence of atrial fibrillation increases with age and more than 1/3 of patients are > 80 years old [9].

The incidence of AF is 88% higher in patients with OSA however that of OSA in AF patients cannot be ascertained as it tends to be severely underdiagnosed in these cases [10].

OSA and AF share many common risk factors encouraging the bidirectional relationship between the two [9]. This overlap of risk factors can also cloud the direct causal relationship between the two. The need to further explore their bidirectional goes beyond atrial fibrillation to include stroke and other cardiovascular comorbidities.

## Methodology

To conduct this review, a literature search was performed across key databases, including PubMed, MEDLINE, Embase, Scopus, and Cochrane Library. The search utilized a combination of Medical Subject Headings (MeSH) terms and relevant keywords focusing on atrial fibrillation, sleep disorders, and sleep-disorder-related complications. Inclusion criteria encompassed studies published in English, spanning from the inception of databases to the present. The review included observational studies, randomized controlled trials, systematic reviews, meta-analyses, and case reports addressing the relationship between atrial fibrillation and sleep disorders. Exclusion criteria involved studies not pertinent to the review's scope or those lacking crucial information on outcomes, management, and safety. Data extraction involved gathering pertinent information from selected studies, including study design, sample size, participant characteristics, intervention details, outcomes, and follow-up duration.

## Main text

### Epidemiology of sleep disorders and atrial fibrillation

Sleep-disordered breathing (SDB) is commonly seen in individuals with atrial fibrillation [11]. The use of polygraphy on 579 patients with paroxysmal AF to test for sleep-disordered breathing yielded significant results [12]. In North America, atrial fibrillation (AF) is the most prevalent chronic arrhythmia, impacting 0.4% of the overall population [13]. Substantial correlations have been shown in earlier research between sleep apnea and AF in patients with congestive heart failure. In 81 congestive heart failure patients, a study found that AF prevalence was considerably higher in individuals who also had sleep apnea than in individuals who did not [14].

Although the researchers established a link between sleep apnea and AF, it remains unclear whether AF was associated with sleep apnea in general or with obstructive sleep apnea (OSA) or central sleep apnea (CSA) specifically. In total, 450 individuals from the Toronto Rehabilitation Institute Sleep Research Laboratory who had congestive heart failure were included in a broader study that also investigated this issue [15]. In that investigation, we discovered a solid connection between AF and CSA, but not OSA. Just 12% of patients with OSA and 7.5% of patients without sleep apnea had AF, but 23% of CSA patients had it. Thus, there is a substantial correlation between AF and CSA in patients with congestive heart failure. The number of people with AF in the USA only is estimated to be between 3 and 6 million, and by 2050, it is expected to rise between 6 and 16 million [16, 17].

In 2010, approximately 9 million people over the age of 55 in Europe had atrial fibrillation (AF), which is about half the population of New York. It is predicted that by 2060, this number will rise to 14 million [18, 19]. Additionally, it was projected that by 2050, about 72 million people in Asia will be diagnosed with AF, which is roughly twice the population of California. Of those cases, approximately 3 million are expected to be associated with strokes [20].

Among patients suffering from OSA, AF may be triggered by intermittent hypoxemia, hypercapnia, chemoreceptor excitation, significantly elevated sympathetic drive, and increased blood pressure. These symptoms may occur nocturnally over a long time if left untreated [21]. Arrhythmogenicity can result from both hypoxemia and hypercapnia [22, 23]. Patients with OSA experience persistent nocturnal elevations in sympathetic activation during wakefulness [24], and AF is linked to elevated sympathetic drive [25, 26]. There are noticeable changes in the size of the cardiac chamber and transmural pressures that occur because of the vigorous ventilatory attempts made to clear the upper airway obstruction during apneas [27, 28].

The stimulation of stretch-activated atrial ion channels by these abrupt structural alterations may facilitate AF [29]. Furthermore, increased levels of C-reactive protein and other markers of systemic inflammation are significantly linked to the severity of OSA [30]. Consequently, there is a clear correlation between rising AF load and C-reactive protein [31]. Interestingly, AF and OSA have a lot of similar risk factors [9]. Thus, common risk factors such as obesity and CVD may be the cause of the link between OSA and AF. This has led to ongoing discussion on whether there is a direct causal relationship between OSA and AF. This is because there is a close correlation between CVD and both disorders. A higher body mass index (BMI) or obesity, advanced age, diabetes, hypertension, smoking, dyslipidemia, and male gender are among the risk factors for these two illnesses [9, 32–34]. According to a recent meta-analysis, ambulatory systolic blood pressure (BP) is a more accurate predictor of future atrial fibrillation (AF) than clinic BP. It was also found that 24 h, daytime, and nighttime systolic BP is all comparable with future AF [35]. Although the exact mechanism underlying how hypertension affects AF is unknown, two primary theories have been put forth.

First, hypertension can lead to hemodynamic abnormalities and an increase in left atrial pressure, which can enlarge the atrium. Secondly, autonomic dysregulation and hypertension-related activation of the renin–angiotensin–aldosterone system (RAAS) can also induce left atrial (LA) fibrosis, which can enhance the development of AF [36]. There are several pathophysiological similarities between OSA and hypertension, including increased fluid distribution, RAAS, gender, obesity, lifestyle, and poor sleep quality [32]. OSA contributes to secondary hypertension because nighttime apneas can raise average systolic and diastolic blood pressure by causing blood pressure to rise. While BMI, diabetes, smoking, dyslipidemia, and sex are known to be predictors of both OSA and AF, they were not shown to have an impact on the association between OSA and AF. There may be pathophysiological relationships between AF and OSA; however, these relationships are still theoretical. Through several pathways, having OSA may cause the onset of AF or exacerbate its consequences [37].

Negative intrathoracic pressure can occur in OSA patients [38]. Both atrial stretch and afterload may rise because of this negative pressure [9]. Consequently, there may be electrical remodeling and left ventricular hypertrophy (LVH), which raise the risk of AF [9]. Smaller refractory and action potential durations, which result from elevated vagal and sympathetic activity and cause structural and electrical remodeling in the atria, are another effect of OSA that may precipitate the onset of AF [39]. Moreover, an increase in inflammation and oxidative stress are brought on by a decrease in O_2_ and an increase in CO_2_, which may potentially aid in the onset of AF [40]. Thus, by distorting the heart's normal electrical activity, OSA may trigger or worsen AF [41–44]. According to this viewpoint, it is possible that treating OSA will help to lessen the symptoms of AF, and research data provide credence to this theory. Surprisingly, a recent meta-analysis revealed that CPAP treatment for OSA is linked to a lower risk of AF recurrence in individuals who did not have electrical cardioversion (ECV) or radiofrequency ablation (RFA) treatment [45].

### Impact of sleep-related autonomic dysregulation and inflammation on atrial fibrillation

Sleep disorders have long been recognized as potential contributors to the development and progression of atrial fibrillation (AF). The connection between sleep disturbances and AF involves multifaceted pathophysiological mechanisms [46]. One such is the autonomic dysregulation caused by sleep disorders and how it plays a huge role in the pathogenesis of AF. Autonomic dysregulation has been demonstrated in several sleep disorders, including obstructive sleep apnea (OSA) and central sleep apnea (CSA) [46].

In OSA, recurrent episodes of upper airway collapse cause intermittent hypoxia and hypercapnia, thereby stimulating a hypoxic stress response by the sympathetic nervous system [47]. This response leads to sympathetic over-activity which persists during brief arousals from sleep, contributing to tachycardia and increased blood pressure [47]. Also, the repetitive apneic episodes are triggers of oxidative stress and inflammation, which can in turn cause endothelial dysfunction that leads to atrial remodeling and subsequent changes favoring AF substrate formation [48]. OSA by itself is directly linked with oxidative stress. Yamauchi et al. conducted a cross-sectional study on the connection between OSA severity and oxidative stress, using a sample size of 128 participants. Fifty individuals had severe OSA (Apnea–Hypopnea Index ≥ 30), while 70 participants had non-severe OSA (Apnea–Hypopnea Index ≤ 30). Urinary levels of 8-hydroxy-2’-deoxyguanosine, a marker of oxidative stress, were significantly elevated among patients with severe OSA [49]. OSA is associated with higher levels of interleukin-6 and tumor necrosis factor-alpha, which is known to enhance a systemic proinflammatory state [50]. This further supports the hypothesis that OSA can cause inflammation in the cardiac endothelial lining. In some animal studies, chronic intermittent hypoxia has been linked to increased atrial fibrosis, shortened refractory period, and increased AF inducibility [51].

The autonomic dysregulation caused by sleep disorders has been shown to have direct acute effects on the electrophysiology of the heart. Impaired central respiratory drive is the hallmark of CSA [52]. Transient withdrawal of parasympathetic tone during episodes of apnea leads to unopposed sympathetic activity. This consequent autonomic imbalance further suppresses vagal tone in the heart and increases sympathetic outflow, promoting a proarrhythmic environment where AF can be kick-started [53]. Hypercapnia by itself slows the velocity of conduction, further contributing to AF susceptibility [54].

Obstructive sleep apnea has been linked to atrial fibrillation development, and a critical pathophysiological mechanism behind this relationship is the concept of negative intrathoracic pressure [55]. During the episodes of upper airway collapse, there is resultant hypoxia, which the body tries to overcome by increasing negative intrathoracic pressure for adequate ventilation. This negative pressure in turn contributes to the pathogenesis of atrial fibrillation [55]. Upper airway obstruction reduces intrathoracic pressure to level below − 50 mmHg [56]. This then causes increased venous return, diminished ventricular filling, and reduced stroke volume. Thus, the circulating blood volume is restricted to the intrathoracic cavity, causing atrial dilatation. Persistent atrial dilatation causes left atrial enlargement, which may stimulate remodeling at the pulmonary vein ostia and subsequently initiate AF [57].

Baroreceptors in the intrathoracic are also activated by negative intrathoracic pressure. This causes a shortening in the refractory period due to an induction of autonomic reflex responses and a subsequent increased AF susceptibility, as demonstrated in a pig model study [58].

Contrary to the idea that increased sympathetic stimulation causes AF in OSA patients, there is a hypothesis linking other arrhythmias with increased parasympathetic activity. During an apneic episode, the vagus nerve is transiently stimulated by increased intrathoracic negative pressure, while the carotid body is stimulated by hypoxemia. Both stimuli cause temporary parasympathetic overactivity, which favors the development of bradyarrhythmias [59].

### Sleep disorders as risk factors for atrial fibrillation

While good sleep is often described as being essential for good health [60] and good health is described as the absence of illness, it is no surprise that the absence or inability to have proper sleep (either in quality or duration) has been discovered to increase the risk of certain cardiac rhythm disorders such as AF [61, 62].

To understand the role of sleep disturbances in AF, we explore the role of sleep duration (with normal sleep being referenced as 8 h), sleep quality, and various sleep disorders associated with AF.

### Sleep duration and atrial fibrillation

Initially, widespread consensus was that low sleep duration (< 6 h) was associated with an increased risk of developing AF [63]. However, with more studies being able to prove the correlation of both low sleep duration and excessive sleep time (> 9 h), it is now a more prevalent notion that abnormal sleep duration and timing of any kind (whether excess or little) could increase the incidence of AF in the affected population [61]. Understanding the effects of sleep duration on AF is becoming increasingly important as some studies outrightly call for the use of sleep duration and sleep quality management in the stratification of AF.

### Sleep quality and atrial fibrillation

While emphasizing that short and long sleep duration may increase AF risk, we must also evaluate the increasing evidence that irregular sleep schedules and below-quality sleep can also play a role in increasing AF risk [64]. The risk of developing AF in patients with abnormal sleep quality such as mild insomnia, sleep apnea, and relative sleep disturbance was all elevated compared to the risks in individuals with normal sleep quality [65].

It is important to note that one proposed mechanism for the relationship between sleep apnea and AF is the resulting hypoxia and hypoxemia that occurs following brief breathless spells. The previous (hypoxemia) refers to reduced blood oxygen levels and the latter (hypoxia), reduced oxygen availability at the tissue level. Hypoxia triggers a cascade that activates the autonomic nervous system resulting in increased heart rate and contractility resulting in elevated vascular resistance. This compensatory mechanism now gives rise to systemic hypertension. Hypertension is a well-documented risk factor for AF [66].

### Specific sleep disorder and atrial fibrillation

While various sleep disorders have been associated with atrial fibrillation, some have been more clinically researched than others. One example of this is obstructive sleep apnea (OSA). OSA is a condition in which the muscles supporting structures such as the tongue, and soft palate temporarily relax. A major risk factor for OSA is obesity [67]. In a recent meta-analysis study, OSA was found to be clinically correlated to an increase in the incidence of developing AF in the diseased population. [68]. It is important to note, however, that while these results are statistically significant, it is not yet determined at what stage of OSA is associated with the highest risk of AF.

Another disease that has been widely clinically documented is restless leg syndrome (RLS). In restless leg syndrome, patients present with an irresistible urge to move their legs or mimic leg motion. It is also commonly associated with iron deficiency [69]. RLS has been shown to increase the risk of AF in diseased populations [70]. A recent study was also able to highlight that RLS could also worsen AF severity from persistent to even permanent forms [70].

One other most recognized sleep disorder associated with AF is Narcolepsy. Narcolepsy is a disorder characterized by prolonged daytime sleepiness, cataplexy, and sleep paralysis [71]. Research has been able to show that since Narcolepsy can cause fragmented sleep, it can decrease the quality of sleep and effectively decrease sleep duration in patients, hence increasing the risk in diseased populations as compared to healthy populace [72].

### Sleep and AF-related risk factors

The bidirectional relationship between both sleep disorders and AF exists in large part due to the shared comorbidities [73]. Certain comorbidities found as a result of sleep disorders are also predisposing risk factors for AF. Chief of which is hypertension. Obesity and smoking are also readily identified [66]. Another example is seen in Narcolepsy. Narcolepsy which is often associated with ANS dysfunction may in turn disrupt normal electrical activity of the heart and trigger AF [74].

Having understood the correlation between sleep disorders and AF, it is important to also emphasize that although such a connection exists, sleep disorders are still very under or misdiagnosed [75]. This could be a missing link to early risk stratification and even early initiation of preventive measures for patients with a high chance of developing AF.

### Implications for risk stratification in patients with both sleep disorders and atrial fibrillation

It is critically important to stratify the risk in patients with atrial fibrillation because of the high risk of mortality and morbidity associated with it. Obstructive sleep apnea is highly associated with atrial fibrillation and affects cardiovascular risk in patients with atrial fibrillation. It has been seen that obstructive sleep apnea (OSA) is more prevalent in patients diagnosed with atrial fibrillation.

2 MACE (Major Adverse Cardiovascular events) was developed for the assessment of cardiovascular risks in patients with atrial fibrillation [76]. There is a direct relationship between the severity of OSA and 2MACE scoring. This indicates the severity of OSA is directly proportional to the increased cardiovascular risk [77].

### Importance of integrated screening approaches in clinical practice

Integrated screening approaches for OSA and atrial fibrillation in clinical practice are important for several reasons. Mostly, OSA and Atrial fibrillation coexists; screening approaches help us in the early diagnosis, leading to early comprehensive care.

It also leads to improved patient outcomes, i.e., reduces cardiovascular risk in such patients. The conditions can be managed accordingly and effectively. OSA can be managed by lifestyle changes, CPAP therapy, or surgery. Atrial fibrillation is treated with anticoagulants, rhythm control strategies, or catheter ablation [78].

Integrated screening can ultimately lead to improved quality of life. This includes better sleep quality, reduced daytime fatigue, and a lower risk of stroke and other heart-related conditions [79].

### Management strategies of AF in patients with sleep disorder

The management principles of atrial fibrillation commonly include rate/rhythm control and anticoagulation which is important as AF serves as a risk factor for developing other clinical conditions such as stroke and heart failure. This condition could also have an impact on the lifestyle and mental health of its sufferers. Therefore, it is important that risk factors and causes are identified and promptly tackled to give these patients a good quality of life.

In this section we look at a few sleep disorders associated with AF and how to manage AF in patients with these sleep disorders. Although sufficient data are lacking in many areas, we hope that soon, there will be certain, more effective management protocols for the AF population with sleep disorders.

### Management of obstructive sleep apnea in patients with atrial fibrillation

1. Continuous Positive Airway Pressure (CPAP) Therapy.
  Continuous Positive Airway Pressure is the most effective treatment for moderate/severe and symptomatic OSA as it keeps the pharyngeal area from collapsing, thereby alleviating airway obstruction. In a randomized study done by Nalliah et al. to evaluate the impact of OSA management on the atrium in AF patients, it was discovered that CPAP therapy reversed atrial remodeling and this could be a potent therapy for rhythm control in this population [80]. It has also been noted that the risk of AF recurrence and progression is increased in OSA patients not using CPAP therapy. Studies have also shown that in patients who have both sleep apnea and atrial fibrillation, the recurrence rates of AF are much higher after a cardiac ablation in patients not using CPAP [81]. Untreated or under-treated sleep apnea is one of the most common reasons for recurrent atrial fibrillation after an ablation. Therefore, CPAP therapy is of great importance in this population.
2. Vagus Nerve Stimulation (VNS).
  A study by Malow et al. showed that VNS during sleep resulted in decreased airflow and reduced respiratory efforts which could precipitate or exacerbate apnea/hypopnea [82]. The heart is innervated by the vagus at both cardiac muscle cells and the conduction system. The effects of vagus nerve stimulation (VNS) can to some extent be predicted from its anatomical distribution. They include a reduction in heart rate (negative chronotropic action on SAN), reduction in atrioventricular conduction (negative dromotropic action on AVN), changes in threshold for induction of atrial fibrillation (AF) (action on atrial conduction system), reduction in ventricular contractility (negative inotropic action on the ventricular myocardium), reduction in threshold for induction of ventricular arrhythmias (action on ventricular conduction system) [83]. Research is still ongoing for the use of VNS in AF. However, a trial carried out by Yu et al. [84] using low-level transcutaneous electrical stimulation (LL-TS) of the auricular branch of the vagus nerve at the tragus in canines suggested that LL-TS repressed the shortening of atrial refractoriness and autonomic remodeling and protected against OSA‐induced AF. More studies need to be carried out on human models to determine if this could be a possible future management.
3. Renal Denervation (RDN).
  A study carried out by Xiakereti et al. [85] on beagle dog models to identify the effect of RDN on OSA-induced AF concluded that RDN reduced renal sympathetic hyperactivity and RAAS activation. It also showed that sympathetic neural remodeling of the substrate AF was downregulated, reducing AF inducibility. In the clinical setting, Witkowski et al. reported that RDN lowered blood pressure in patients with refractory HT and OSA, which was accompanied by an improvement in sleep apnea severity [86].
  Based on studies that have shown that RDN could reverse the structural and sympathetic remodeling of the atrium, it is safe to say that this could become a novel modality for managing AF in OSA patients shortly.
4. Low-level Baroreflex stimulation.
  Baroreceptor stimulation not only modulates autonomic balance by sympathetic withdrawal but also increases vagal activation; therefore, it has been valuable in treating patients with heart failure and resistant hypertension [87]. High-frequency baroreceptor stimulation could reduce sinus rate and increase chances of AF inducibility; however, a stimulation voltage set at about 80% below the sinus rate reducing threshold can increase the atrial effective refractory period and AF threshold, thereby suppressing AF inducibility in dog models [88]. This study shows that LL-BRS but not HL-BRS could be therapeutic for AF associated with OSA.
5. Beta-Blockers.
  Beta-adrenergic antagonists are commonly used medications in managing AF for rate control. They also help in controlling ventricular rate and reducing the risk of mortality. It is the preferred rate control agent for patients with AF following a myocardial infarction and for patients with congestive heart failure [89].
  The reported benefits of beta-blocker use in CVD patients with concomitant obstructive sleep apnea (OSA) might be limited by the thoughts of beta-blockers aggravating the apnea-induced bradycardias that are observed in these patients. However, a study negated this thought by showing that beta-blockers not only reduce apnea-induced increases in heart rate, but they do not potentiate apnea-induced HR decelerations as well as have no influence on respiratory disturbances [90]. Evidence brought by a study done on canine models of chronic OSA where metoprolol (5 mg/kg/day) was continuously administered showed inhibition of structural, sympathetic neural, and metabolic remodeling in the atria and this might potentially play a role in treating atrial remodeling, decreasing AF inducibility, and shortening the duration of AF [91]. This might be a potent and cost-effective therapy for managing AF in people with OSA; hence, future trials should be attempted on humans.
6. Weight Loss.
  There is a proven link between obesity and atrial fibrillation. There are multiple studies demonstrating the relationship between increasing BMI/obesity and AF. A study by Wang TJ et al. [92] showed that regardless of gender, for every 1% increase in BMI, there was a 4% increase in AF risk. Likewise, obesity is probably the most significant yet modifiable risk factor for developing obstructive sleep apnea. In most cases, weight loss may be an effective therapy for overweight/obese people with OSA; this could reduce the symptoms and sometimes lead to resolution of OSA [93]. Larger cohorts such as one conducted by Huxley et al. 2011 [94] involving 14,598 participants with a mean follow-up period of 17 years also support this, as 17.9% with atrial fibrillation was attributed to overweight and obesity.
  Additionally, Tedrow et al. 2010 [95] also conducted a prospective cohort with a follow-up period of 12 years involving 34,309 female participants showed a 4.7% increase in atrial fibrillation per 1 kg/m^2^ increase in BMI.
  Tsang et al. 2008 [96] in another prospective cohort study among 3,248 patients with a paroxysmal form of atrial fibrillation concluded that BMI predicted progression of paroxysmal to permanent AF with a hazard ratio of 1.04.
  If sleep apnea is managed properly, there is a possibility that atrial fibrillation may go away. This makes it important for lifestyle modifications and weight loss strategies to be implemented in managing atrial fibrillation due to sleep disorders.

### Challenges and future directions

Atrial fibrillation patients may experience sleep disorders differently than those without AF. Screening tests like sleep questionnaires and polysomnography (PSG) derived from the general medicine practice do not work well in patients with cardiovascular diseases such as atrial fibrillation. Patients with atrial fibrillation do not show the exact pathognomonic signs such as snoring or daytime sleepiness. The other symptoms such as fatigue and nocturnal dyspnea can be multifactorial such as caused by underlying comorbidities [97]. Further research is needed to evaluate sleep disorders in patients with cardiovascular diseases.

Limited access to sleep specialists; specialized sleep centers may not be readily accessible, particularly in rural or underserved areas. This can make it difficult for AF patients to receive comprehensive evaluation and treatment for sleep disorders.

Obesity is accompanied by various medical conditions such as hypertension, diabetes mellitus, and obstructive sleep apnea. Hypersthenic posture can lead to sleep apnea, which then causes AF. In this group of patients, weight loss can improve the prognosis of AF [98].

Hang Dinh Thi Dieu et al. conducted a prospective and descriptive study on 139 individuals with severe OSA with auto-adjusting CPAP at home for 6 months. The patients were followed up for over 6 months. The patients showed some side effects in the 3rd and the 6th month. The main side effects in the 3rd month were dry mouth or nose, difficulty sleeping, skin marks or rashes, and discomfort breathing. In the 6th month, some patients refused to use the CPAP due to uncomfortable feelings while sleeping [99]. However, further research is needed to improve the CPAP device without causing side effects to the patients.

Future investigations on sleep disorders in atrial fibrillation patients are on molecular and cellular levels. Neural modulation (autonomic) therapy is one of the future directions in treating OSA-associated atrial fibrillation patients [87].

Randomized control trials are needed to assess the effectiveness and duration of various sleep therapies in preventing atrial fibrillation recurrence and improving overall health.

## Conclusions

This narrative study concludes sleep disorders are noted to have a bidirectional relationship with atrial fibrillation. There is a strong association between sleep disorders and atrial fibrillation patients with sleep disorders such as obstructive sleep apnea (OSA) which is more likely to develop atrial fibrillation and those with atrial fibrillation are more likely to have sleep disorders. Sleep disorders can contribute to atrial fibrillation development and progression, and atrial fibrillation can worsen the quality and duration of sleep. Patients with CPAP have proved to have side effects and worsen the quality of sleep due to the continuous usage of the device during the night.

Sleep disorders may play a major role in the atrial remodeling and electrophysiological abnormalities of the heart that contribute to atrial fibrillation. Sleep disorders may also alter the structure and function of atrial tissue which makes it more susceptible to arrhythmogenesis. This narrative review has shown that treating sleep disorders may improve sleep quality and reduce the risk factors of atrial fibrillation. Treating the sleep disorder can be an effective challenge for atrial fibrillation prevention and management.

Patients with atrial fibrillation and sleep disorders should be educated about the importance of sleep and the benefits of the treatment for sleep disorders. This narrative review also explosively emphasizes the importance of the diagnosis of sleep disorders in atrial fibrillation patients.

## Data Availability

Data sharing is not applicable to this article as no datasets were generated or analyzed during the current study.

## Abbreviations

AF: Atrial fibrillation
BMI: Body mass index
CSA: Central sleep apnea
CVD: Cardiovascular disease
CPAP: Continuous positive airway pressure
ECV: Electrical cardioversion
HL-BRIS: High-level baroreceptor stimulation
LL-BRS: Low-level baroreceptor stimulation
OSA: Obstructive sleep apnea
RAAS: Renin angiotensin aldosterone system
RFA: Radio-frequency ablation
SDB: Sleep-disordered breathing
VNS: Vagus nerve stimulation
